# Associations of Work-Family Conflict with Family-Specific, Work-Specific, and Well-Being-Related Variables in a Sample of Polish and Ukrainian Adults during the Second Wave of the COVID-19 Pandemic: A Cross-Sectional Study

**DOI:** 10.3390/ijerph191710954

**Published:** 2022-09-02

**Authors:** Cezary Kuśnierz, Aleksandra M. Rogowska, Karolina Chilicka, Iuliia Pavlova, Dominika Ochnik

**Affiliations:** 1 Faculty of Physical Education and Physiotherapy, Opole University of Technology, 45-758 Opole, Poland; 2 Institute of Psychology, University of Opole, 45-040 Opole, Poland; 3 Department of Health Sciences, University of Opole, 45-040 Opole, Poland; 4 Department of Theory and Methods of Physical Culture, Lviv State University of Physical Culture, 79007 Lviv, Ukraine; 5 Faculty of Medicine, University of Technology, 40-555 Katowice, Poland

**Keywords:** anxiety, COVID-19 pandemic, depression, gender, parenthood, perceived stress, time pressure, relationship status, remote work, work-family conflict

## Abstract

The conflict between work and family demands increased during the COVID-19 pandemic due to changes in lifestyle related to the lockdown. This study examines the associations between work-family conflict (WFC) and family-work conflict (FWC) with work-specific, family-specific, and well-being-related variables during the second wave of the COVID-19 pandemic. The results may be used in practice to improve the well-being of employees by adjusting home-based work and family areas of life to dynamic changes during the pandemic. The sample of 736 adults from Poland (53.26%) and Ukraine (46.74%), aged between 19 and 72 (*M* = 39.40; *SD* = 10.80), participated in the study. The cross-sectional study was performed using an online survey, including sociodemographic variables, measures of WFC, time pressure, remote work assessment (RWAS), physical health (GSRH), life satisfaction (SWLS), perceived stress (PSS-10), anxiety (GAD-7), and depression (PHQ-9). This study showed numerous inter-group differences in all variables across the country, gender, relationship status, parenthood, caring for children under 12, and remote working status. A high WFC is more likely among Polish workers (than Ukrainian workers), people with a low level of self-perceived time pressure, and high symptoms of stress. Caring for children under 12, low self-perceived time pressure, and high stress can predict FWC. Various paths lead from perceived stress via WFC and FWC, physical health, anxiety, and depression to life satisfaction, as suggested by the structural equation modeling analysis. Parents of children under 12 and women are the most vulnerable groups for increased WFC, FWC, and worse mental health and well-being. Prevention programs should focus on reducing stress, anxiety, and work demands in these adult populations. A unique contribution to the existing knowledge revealed patterns of associations between WFC and FWC in relation to well-being dimensions in a cross-cultural context during the pandemic.

## 1. Introduction

Family and work play a central role in current adult life. However, dependent on priorities and importance, people may differ in their assessment of these two domains [1]. A work-family conflict occurs when work-related stress, work engagement, demands, and overload interfere with family life, disrupting the social roles of a spouse, partner, and parent. In family-to-work conflicts, commitments to family life (e.g., caring for young or disabled children or elderly parents, conflicts with adolescents or spouses, lack of support from family members) adversely affect work quality or producibility, career, and aspirations. The conflict between work and family can include competing time requirements (time-based), impaired performance (burden-based), and behavioral incompatibility (behavior-based) between work and family roles. Work-family balance requires continuous negotiations between partners regarding shared role-related expectations and accomplishments [2]. A balance between work and family increases life satisfaction and the quality of life [2,3,4,5]. In contrast, work-family conflict can lead to several adverse consequences, such as disrupting one’s professional career and family cohesion, decreasing work satisfaction and performance, and low levels of physical and mental health and well-being in family members [6,7,8]. In particular, work-to-family conflict (WFC) decreases family satisfaction, while family-to-work conflict (FWC) decreases job satisfaction, and both job and family satisfaction contribute to balancing one’s work and family life [9].

There are several sources of WFC, including temporal aspects of work (the number of working hours, travel time, and overtime) and its stressful properties (high physical and mental demands, conflict and role ambiguity, pace of changes in the work environment, and low social support). In contrast, primary sources of FWC are related to marital status, family size, childcare, a spouse’s professional burdens, and support from the partner and other family members [10]. Time pressure in the work domain may include working hours, an inflexible work schedule, and shift-work, leading to greater strain and role conflict (based on role ambiguity and boundary-spanning activities) and changing one’s behavior relating to expectations for secretiveness and objectivity. Regarding the family domain, time pressure is correlated with caring for young children and spouse employment, and a large family may increase strain and family conflict (especially when spouse support is low), changing one’s behavior depending on expectations for warmth and openness. The Greenhaus and Allen [11] model of work-family balance supposes that work interference with family (WIF) is predicted by work experience (work involvement and work role characteristics) and dispositional factors (such as personality traits). In contrast, family interference with work (FIW) is predicted by family experiences (family involvement and family role characteristics) and dispositional factors. Interaction between WIF and FIW can change the effectiveness and satisfaction of work and family, contributing to a work-family balance.

Research indicates that WIF and FIW are dependent on cross-cultural and gender differences. Meta-analysis showed that people from more developed countries (individualistic) are more sensitive to WIF than those from less developed countries (collectivistic) [12,13]. Poland and Ukraine are adjacent to each other in Europe, sharing a similar history, the transition from communism to democracy, and the experience of being threatened by Russia. However, Poland currently belongs to the European Union, while Ukraine aspires to join the EU, fighting against the invasion of Russian troops. Ukraine belongs to developing countries and was ranked as the poorest country in Europe in 2020, since the gross national income (GNI) per capita was 3540 USD. The economic status in Poland is below the European average but is higher than that in Ukraine by fivefold (GNI per capita was 15,656 USD). Therefore, it is interesting to see whether the two countries differ in mean levels of WIF and FIW.

The direction of role interference in WFC-FWC may differ by gender. A traditional gender role ideology (GRI) believes that family should be a woman’s priority, while work should be a man’s responsibility. In contrast, non-traditional or egalitarian GRI assumes that women and men should distribute their roles equally for work and family [14]. The research found the interaction between culture and gender, with greater WIF and FIW, reported in countries with more traditional than egalitarian gender role beliefs [15]. The systematic review [16] suggests changing the expectations and practices around work-family balance due to current cultural changes in the technological revolution, job insecurity, family diversity, and new masculinities. Therefore, more research on work-family balance is required from a cross-cultural and gender comparative perspective. 

The coronavirus disease of 2019 (COVID-2019) significantly impacted current life, changing lifestyle and behavior in family and work domains. The COVID-19 pandemic also changed the overall work organization and arrangements, accelerating and disrupting various trends in the work area [17]. In particular, many schools and workplaces were closed during the lockdown, so stationary work was changed to remote online, and working parents were forced to divide their time between caring for children and teleworking [18,19,20,21,22,23,24,25]. Experts assumed the transition to telework was one of the essential issues for occupational health during the COVID-19 pandemic [26]. Studies report that occupational stress increased alongside WFC and FCW during the pandemic, especially among teleworkers [20,21,27,28,29,30,31]. Among people working remotely, WFC and FWC were associated with adverse physical symptoms, high stress, anxiety, depression, burnout, and low life satisfaction [27,28,31,32,33]. 

The imbalance between family and work responsibilities has increased during the COVID-19 pandemic, as suggested by a recent systematic review [16]. One of the most sensitive groups for high WFC and FWC was parents of minor children [22,23,24]. For example, a disturbed work-family balance was found among German medical doctors due to insufficient childcare when schools and preschools were closed during the pandemic [19]. The study among Portuguese-employed parents showed that, dependent on the stationary or online type of work, diverse experiences were reported during the lockdown [18]. Those working online at home reported higher levels of stress and anxiety and more difficulties in co-parenting than those working stationary (out-of-home) and unemployed. 

Women with fewer educational, economic, and professional resources were more likely to play the role of caregivers, contributing to gender inequalities at work and in the family, as suggested by the literature [16]. Compared to men, women were more responsible for childcare, schooling, and household tasks, working primarily from home and reducing their work hours [19,23,34,35,36,37]. Overload in caregiving and overall workload in women was related to heightened WFC and FWC, as well as parenting stress, general perceived stress, concerns about job insecurity, general and neck/shoulder pain, stress, depressive and anxiety symptoms, and lower life satisfaction [19,23,24,34,35,36,37,38,39,40,41,42,43]. In particular, solely employed mothers were at higher risk of mental health adverse effects and job-related well-being than partnered working mothers [38]. 

### The Purpose of the Current Study

This study aims to examine associations between WFC and FWC, selective job-related variables (seniority, remote vs. stationary job type, remote working assessment), family-related factors (time pressure, relationship status, having children, especially those aged below 12), and well-being dimensions (physical health, stress, anxiety, depression, and life satisfaction). The complex model of the relationships between WFC, FWC, and well-being dimensions will be explored for the first time, to our best knowledge, in a cross-cultural context during the pandemic. Based on the stress-strain model [44], all pandemic-related variables are considered stressors, and the work-family conflict is a strain. The Contextual Model of Family Stress (CMFS) conceptualizes the family as a complex system that assumes interactions between family members and the environment [45,46]. A disruption to the structural and psychological context of the family can weigh on its well-being, increasing strain and conflict between family and work. 

Based on previous literature [17,18,19,20,21,22,23,24,25,26,27,28,29,30,31,32,33], we assume that changes in lifestyle during the COVID-19 pandemic were stressful events that contributed to the work-family imbalance, leading to high WFC and FWC [16]. We will examine the differences in WFC, FWC, well-being dimensions, and time pressure across countries, genders, relationship status, parenthood experience, caring for children under 12, and type of work (stationary vs. remote). We hypothesize that higher CWF and worse well-being will be presented in Poland than in Ukraine regarding the individualism-collectivism dimension [12,13], among teleworkers [20,21,27,28,29,30,31,32,33], women [16,19,23,24,34,35,36,37,38,39,40,41,42,43], coupled individuals, and those having children [16,18,19,20,21,22,23,24,25]. Inter-group differences in time pressure will be explored for the first time in this study. Therefore, we do not assume any direct hypothesis.

The associations between WFC and FWC and well-being dimensions will be investigated using Pearson’s correlation, multiple linear regression, and structural equation modeling (SEM) analyses. According to the stress-strain model [44] and previous studies [20,22,27,28,31,32,33], we assume that high stress during the pandemic can predict adverse consequences, such as increased WFC, FWC, anxiety, depression, and worsening physical health and life satisfaction. However, consistent with the CMFS [45,46], mental health dimensions (i.e., physical health, anxiety, and depression) can mediate between stress and life satisfaction, as well as between family and work conflicts and life satisfaction. 

## 2. Materials and Methods

### 2.1. Procedure

A cross-sectional study was performed in Poland (between 19 November 2020 and 15 January 2021) and Ukraine (between 14 December 2020 and 17 February 2021) during the second wave of the COVID-19 pandemic. The online survey was created using Google Forms and disseminated using the snowball technique. The invitation to research with a link to the survey was sent to friends and private groups on Facebook. Additionally, with the consent and support of the university authorities, the invitation to the study with a link to the survey was sent to all administrative staff and academic teachers by the university’s internal mail, where the authors of the study were employed (i.e., at the Opole University of Technology, the University of Opole, the University of Technology in Katowice from Poland, and Lviv State University of Physical Culture from Ukraine). University authorities showed great support and understanding for the purpose of the study and were also interested in feedback and research conclusions to improve the well-being of workers during the COVID-19 pandemic. The survey included informed consent, sociodemographic questions, and standardized questionnaires to measure work-family conflict and well-being dimensions (physical health, perceived stress, anxiety, depression, and life satisfaction). Participants voluntarily and anonymously (without financial compensation) took part in the survey if they gave informed consent. Among 743 people who answered the invitation, seven refused the study, so the final sample consisted of 736 people. No missing data were reported as all replies were mandatory in the Google form. 

### 2.2. Measurement

#### 2.2.1. Work-Family Conflict 

Carlson et al. [47] developed the multidimensional measure of work-family conflict to measure its six dimensions in the combination of three forms (time, strain, and behavior) with two directions of conflict: work interference with family (work-family conflict, WFC) and family interference with work (family-work conflict, FWC). Each subscale (WFC time, WFC strain, WFC behavior, FWC time, FWC strain, and FWC behavior) consists of three items, to which the participant answers on a five-point Likert scale (from *Completely disagree* = 1 to *Completely agree* = 5). The total scores of WFC and FWC subscales ranged from 9 to 45, with higher levels interpreted as greater conflict. The reliability of the scales (Cronbach’s α) was 0.90 for both WFC and FWC.

#### 2.2.2. Time Pressure

Time pressure was measured using a questionnaire developed for the study’s aim. The questionnaire consisted of 15 items. A participant was asked: “During the last week, how much time did you spend on the following activities during an average day?” The list of 15 items included: 1. shopping; 2. cleaning; 3. cooking, food preparation; 4. childcare; 5. caring for the elderly and/or disabled people; 6. repairs and renovations; 7. social meetings; 8. entertainment, games, and fun; 9. hobbies, development of interests; 10. personal and spiritual development; 11. relaxation and rest; 12. sleep; 13. work in stationary mode; 14. working in remote mode; and 15. learning and training. The answer to each item was rated on a 6-point Likert scale, indicating the amount of time (*Not at all* = 0; *Less than 1 h* = 1; *1–2 h* = 2; *2–3 h* = 3; *3–4 h* = 4; *5 h or more* = 5). Time pressure was rated twice: (1) as a self-assessment (time pressure actor, TPA) and (2) as a perceived appraisal of the partner (time pressure partner TPP). The higher sum of item scores (ranging from 0 to 75) indicates greater time pressure during an average working day. The ordinal reliability of the total score was Cronbach’s α = 0.68 for TPA (average inter-item correlation, *r* = 0.15), and α = 0.79 for TPP (*r* = 0.22).

#### 2.2.3. Remote Work Assessment

Remote work assessment scale (RWAS) was developed in this study to measure the quality of telework during the pandemic. We created ten items: 1. I feel fully trained and prepared to work remotely; 2. Remote work is a source of stress for me; 3. When working remotely, I have unlimited access to a computer or other equipment necessary for my work; 4. Remote work significantly reduces the quality of my work; 5. I have a good internet connection, allowing me to work remotely without problems; 6. I have the appropriate software that will enable me to work remotely; 7. system overload does not affect my remote work; 8. remote work’s effectiveness is significantly lower than stationary work; 9. I have sufficient technical support to resolve computer or software problems regularly; and 10. I prefer to work stationary than remotely. Participants rated on a 5-point Likert scale the agreement with a given item (from *Completely disagree* = 1, to *Completely agree* = 5). Questions 2, 4, 8, and 10 were reversed before summarizing all scores. The total score ranged from 10 to 50, with higher scores indicating better resources, support, remote working experiences, and a more positive attitude towards teleworking. The scale’s internal consistency was Cronbach’s α = 0.82 in the study sample. 

#### 2.2.4. Perceived Physical Health

DeSalvo et al. [48,49] developed the general self-rated health (GSRH) systam as a brief measure of health-related quality of life. The GSRH includes two items derived from the standard general health survey (SF-12 V). Participants rated on the 5-point Likert scale (from *Excellent* = 1 to *Poor* = 5) their health individually (item 1) and in comparison to others the same age as them (item 2). Higher scores are interpreted as worse perceived health. The reliability coefficient in the study was Cronbach’s α = 86.

#### 2.2.5. Life Satisfaction

Diener et al. [50] developed the satisfaction with life scale (SWLS) to assess the global cognitive aspect of subjective well-being. The SWLS consists of five items with a 7-point Likert scale of response (*Strongly disagree* = 1, while *Strongly agree* = 7). The scores range from 5 to 35, and higher scores represent a more heightened satisfaction with life. The internal consistency of the SWLS in the current study was Cronbach’s α = 0.88. 

#### 2.2.6. Perceived Stress

Cohen et al. [51] developed the 10-item perceived stress scale (PSS-10) to assess stressful life events. The PSS-10 describes the frequency of stressful situations in the past month. Participants rate their responses on a 5-point Likert scale (from *Never* = 0, to *Very often* = 4). The total score is a sum of responses for every ten items (ranging from 0 to 40), and higher scores indicate a higher stress level. The internal consistency of the PSS-10 was Cronbach’s α = 0.87.

#### 2.2.7. Anxiety

Spitzer et al. [52] developed the 7-item generalized anxiety disorder (GAD-7) scale as a screening tool to measure anxiety symptoms. The GAD-7 is a short tool for a clinical assessment of anxiety risk during the last two weeks. Participants responded to each of the seven items on how often they experienced anxiety symptoms in the past two weeks, using a 4-point Likert scale (from *Not at all* = 0 to *Nearly every day* = 3). Higher scores (ranging from 0 to 21) indicate more severe anxiety symptoms. The Cronbach’s α in the present sample was 0.94. 

#### 2.2.8. Depression

Kroenke et al. [53] developed the 9-item patient health questionnaire (PHQ-9) to assess depression risk in the general population. Participants answer how frequently each symptom of depression occurred during the past two weeks using a 4-point Likert response scale (from *Not at all* = 0 to *Nearly every day* = 3). The higher the scores (ranging from 0 to 27), the higher the depression risk. The reliability coefficient in the present study was Cronbach’s α = 0.92.

#### 2.2.9. Sociodemographic Variables

The demographic questions regarded country (Poland = 0, Ukraine = 1), age (years), gender (man = 0, woman = 1), relationship status (coupled = 0, single = 1), parenthood experience (childless = 0, parent = 1), caring for children under 12 (no = 0, yes = 1), type of work (stationary = 0, remote = 1), and work seniority (years).

### 2.3. Participants

The sample of 736 adults from Poland (*n* = 392, 53.26%) and Ukraine (*n* = 344, 46.74%), aged between 19 and 72 (*M* = 39.40; *SD* = 10.80) participated in the study. Among them, 486 (66.03%) were women, 155 (21.06%) were single, 502 (68.21%) were parents, and 276 (37.50%) parents had children below 12 years of age. Remote working reported 530 (72.02%) people. Work experience (seniority) ranged between 0 and 53 years (*M* = 15.54, *SD* = 10.34). 

### 2.4. Statistical Analysis

The preliminary analysis regarded descriptive statistics for continuous variables, including scales, such as WFC, FWC, RWAS, multitasking (actor and partner), SWLS, GSRH, PSS-10, GAD-7, and PHQ-9. All scales ranged in skewness between −0.54 and 0.95 and kurtosis between −0.35 and 0.49, indicating appropriate properties for parametric tests, considering a quite large sample size (*N* = 736). Therefore, independent samples student’s *t*-test (with Cohen’s *d* as an effect size) was performed to examine differences in all continuous variables (WFC, FWC, remote work assessment, time pressure, life satisfaction, physical health, perceived stress, anxiety, and depression) between such groups as the country (Poland, Ukraine), gender (women, men), relationship status (single, coupled), parenthood (parents, childless), caring for children under 12 (yes, no), and type of work (remote, stationary). If the inter-group variance was not equal, the Welsh *t*-test was conducted instead of a student’s *t*-test. The sensitivity analysis was performed with a Mann–Whitney *U*-test and ranked biserial correlation (RBC) as an effect size to check the same group differences for the 15-item time pressure scale. In addition, the Kruskal–Wallis *t*-test was conducted for dependent samples to compare actor and partner assessments of time pressure in the coupled sample (*n* = 581). The effect size for the Kruskal–Wallis *t*-test was calculated by dividing the absolute standardized test statistic *Z* by the square root of the number of compared pairs. A three-way ANOVA was performed for WFC and FWC as repeated measures of continuous dependent variables and two dichotomous categorical factors: gender (women, men) and caring for children under 12 (yes, no), with partial eta-square (η^2^*_p_*) as an effect size. The Bonferroni posthoc tests were conducted to examine significant differences between gender and parenthood groups in the mean values of conflict between work and family. 

The associations between variables were assessed using Pearson’s correlations. The multiple linear regression analysis was performed for WFC and FWC separately, with a set of variables, including sociodemographic (country, gender, age, relationship status, parenthood, and caring for children below 12 years of age), work-related variables (work seniority, working remotely, remote work assessment, TPA, and TPP), and well-being dimensions (life satisfaction, physical health, perceived stress, anxiety, and depression). However, when checking whether the assumptions for using multiple linear regression were met, multi-collinearity was found between age and seniority, actor and partner time pressure, and anxiety, stress, and depression. Therefore, we removed age, multitasking partner, and anxiety from the regression model. All statistics were appropriate for the WFC and FWC model, respectively, including the Durbin–Watson statistic (autocorrelation was −0.016 and −0.026, DW was 2.03 and 2.05, the *p*-value was 0.794 and 0.598), multi-collinearity with suitable tolerance (ranging between 0.42 and 0.96 for both WFC and FWC) and variance inflation factor (VIF ranged between 1.05 and 2.39 for both WFC and FWC), multi-variate normality (Kolmogorov–Smirnov was 0.038 and 0.055, *p*-value 0.503 and 0.118), and heteroskedasticity (Breusch–Pagan statistic was 15 and 11, *p*-value 0.305 and 0.613).

Finally, the SEM with maximum likelihood (ML) estimation method was performed for a path model. Life satisfaction was considered in this model as an outcome, while stress, WFC, FWC, physical health, perceived stress, anxiety, and depression as predictors. Multiple mediation analysis was tested using bootstrap techniques with 1000 resampling and bias-corrected (BC) percentile method with 95% confidence intervals. The SEM was evaluated using such goodness-of-fit criteria as maximum likelihood (ML) χ^2^, *df* and *p*-value (the ratio χ^2^/*df* < 5 means good fit), standardized root mean squared residual (SRMR < 0.08 is acceptable), root mean square error of approximation (adequate fit if RMSEA ≤ 0.08), and comparative fit index (CFI ≥ 0.90 meaning adequate fit) [54]. The configural measurement invariance across countries, genders, relationship status, parenthood, caring for children under 12, and remote working were examined using multi-group structural equation modeling (MGSEM). For an adequate sample size (*N* > 300), Chen [55] suggests a change of >−0.010 in CFI, supplemented by a change of >0.015 in RMSEA or a change of >0.030 in SRMR, which suggests non-invariance. Descriptive statistics and correlation were performed using JASP software (JASP Team, Amsterdam, The Netherlands, 2020)) for Windows ver. 0.16.1, multiple linear regression with JAMOVI (The JAMOVI Project, Sydney, Australia, 2022) for Windows ver. 2.2.5.0, and SEM model was conducted using AMOS ver. 26 and IBM SPSS ver. 26 (IBM Corp., Armonk, NY, USA, 2019) for Windows.

## 3. Results

### 3.1. Group Differences in Work-Related Variables and Well-Being Dimensions

Differences between groups were examined using independent samples student’s *t*-test. Country differences are shown in Table S1 (in Supplementary Materials). Polish adults presented significantly higher scores in FWC (*p* < 0.001, *d* = 0.33), remote work assessment (*p* = 0.019, *d* = 0.24), perceived partner’s time pressure (*p* = 0.022, *d* = 0.17), perceived stress (*p* < 0.001, *d* = 0.28), and anxiety (*p* = 0.003, *d* = 0.22), compared to Ukrainian participants. Gender differences are presented in Table S2. Women scored significantly higher than men in WFC (*p* = 0.020, *d* = −0.18), stress (*p* < 0.001, *d* = −0.28), anxiety (*p* < 0.001, *d* = −0.32), and depression (*p* < 0.001, *d* = −0.33), and they have worse physical health (*p* = 0.007, *d* = −0.21). Regarding relationship status (Table S3), single participants showed statistically significant lower life satisfaction in comparison to coupled people (*p* < 0.001, *d* = 0.43). Compared to childless individuals (Table S4), parents assessed significantly lower remote work (*p* = 0.022, *d* = −0.33) and self-time pressure (*p* = 0.001, *d* = −0.25), while partner’s time pressure was higher (*p* < 0.001, *d* = 0.37), and they demonstrated worse physical health (*p* = 0.011, *d* = 0.21), but higher life satisfaction (*p* < 0.001, *d* = 0.32). Parents caring for children under 12 scored significantly higher than those without small children in WFC (*p* < 0.001, *d* = −0.29), FWC (*p* < 0.001, *d* = −0.47), and perceived partner’s time pressure (*p* < 0.001, *d* = −0.36), and they presented greater symptoms of stress (*p* = 0.005, *d* = −0.21), anxiety (*p* = 0.003, *d* = −0.23), and depression (*p* = 0.005, *d* = −0.22), which is shown in detail in Table S5. Remote working participants assessed both self (*p* = 0.003, *d* = 0.24) and partner’s (*p* = 0.025, *d* = 0.19) time pressure higher than those working stationary, and they presented significantly lower level of perceived stress (*p* = 0.006, *d* = −0.22), as it is demonstrated in Table S6.

As a sensitivity analysis, particular dimensions of self-perceived time pressure were examined across groups (Tables S7–S13 in Supplementary Materials) using Mann–Whitney *U*-test. Country differences are shown in Table S7. People from Poland spent significantly more time on shopping (*p* = 0.002, RBC = 0.12), cleaning (*p* < 0.001, RBC = 0.14), caring for elderly or disabled people (*p* = 0.013, RBC = 0.09), repairs and renovations (*p* < 0.001, RBC = 0.22), entertainment (*p* < 0.001, RBC = 0.39), hobbies (*p* = 0.035, RBC = 0.09), and stationary work (*p* = 0.008, RBC = 0.11), while participants from Ukraine were more engaged in social meetings (*p* < 0.001, RBC = −0.14), development (*p* < 0.001, RBC = −0.23), and remote work (*p* < 0.001, RBC = −0.29). Gender differences in self-perceived time pressure are presented in Table S8. Women were busier than men with cleaning (*p* < 0.001, RBC = −0.22), cooking (*p* < 0.001, RBC = −0.34), childcare (*p* < 0.001, RBC = −0.21), and remote working (*p* < 0.001, RBC = −0.20), while men spent more time than women on repairs and renovations (*p* < 0.001, RBC = 0.43), social meetings (*p* < 0.001, RBC = 0.15), entertainment (*p* < 0.001, RBC = 0.29), hobbies (*p* < 0.001, RBC = 0.17), relaxation and rest (*p* < 0.001, RBC = 0.18), stationary work (*p* = 0.002, RBC = 0.14), and also learning and training (*p* < 0.001, RBC = 0.17). Differences in self-perceived time pressure between single and coupled individuals are shown in Table S9. Coupled people spent significantly more time than singles on childcare (*p* < 0.001, RBC = 0.40), and remote work (*p* = 0.006, RBC = 0.14), while much less on social meetings (*p* < 0.001, RBC = −0.17), hobbies (*p* = 0.032, RBC = −0.11), and development (*p* = 0.013, RBC = −0.13). 

Additionally, parenthood experiences deferred self-rated time pressure, as shown in Table S10. Parents spent significantly less time than childless participants on shopping (*p* = 0.034, RBC = −0.09), repairs and renovations (*p* = 0.020, RBC = −0.09), social meetings (*p* < 0.001, RBC = −0.24), entertainment (*p* < 0.001, RBC = −0.30), hobbies (*p* < 0.001, RBC = −0.28), development (*p* = 0.028, RBC = −0.10), relaxation and rest (*p* < 0.001, RBC = −0.27), sleep (*p* = 0.020, RBC = −0.08), stationary work (*p* < 0.001, RBC = −0.15), learning and training (*p* < 0.001, RBC = −0.25). In contrast, parents were significantly more engaged in caring for children (*p* < 0.001, RBC = 0.74), elderly, and disabled people (*p* = 0.010, RBC = 0.09), and remote work (*p* = 0.003, RBC = 0.13), compared to childless individuals. As presented in Table S11, parents with children under 12 spent significantly more time caring for children (*p* < 0.001, RBC = −0.85), as well as the elderly and disabled people (*p* = 0.040, RBC = −0.07), compared to people without small children. Parents of children under 12 were less engaged in social meetings (*p* < 0.001, RBC = 0.18), hobbies (*p* < 0.001, RBC = 0.23), development (*p* < 0.001, RBC = 0.25), relaxation and rest (*p* < 0.001, RBC = 0.27), sleep (*p* = 0.006, RBC = 0.09), and also learning and training (*p* < 0.001, RBC = 0.19). 

Differences in time pressure between representing the remote and stationary types of work are shown in Table S12. Remote workers spent less time than stationary workers on childcare (*p* < 0.001, RBC = −0.18), but significantly more time on development (*p* < 0.001, RBC = 0.18), and also learning and training (*p* < 0.001, RBC = 0.28). Finally, differences between actor and partner perceived assessment of time pressure were examined using dependent samples in a Kruskal–Wallis *T*-test (Table S13). Participants assessed themselves significantly higher than their partners in cleaning (*p* < 0.001, RBC = 0.25), cooking (*p* < 0.001, RBC = 0.40), childcare (*p* < 0.001, RBC = 0.36), hobbies (*p* = 0.009, RBC = 0.11), development (*p* < 0.001, RBC = 0.18), relaxation and rest (*p* < 0.001, RBC = 0.16), remote work (*p* < 0.001, RBC = 0.46), and learning and training (*p* < 0.001, RBC = 0.42).

### 3.2. Interaction between the WFC and FWC Conflict, Gender, and Caregiving for Children under 12 

**Figure 1:**
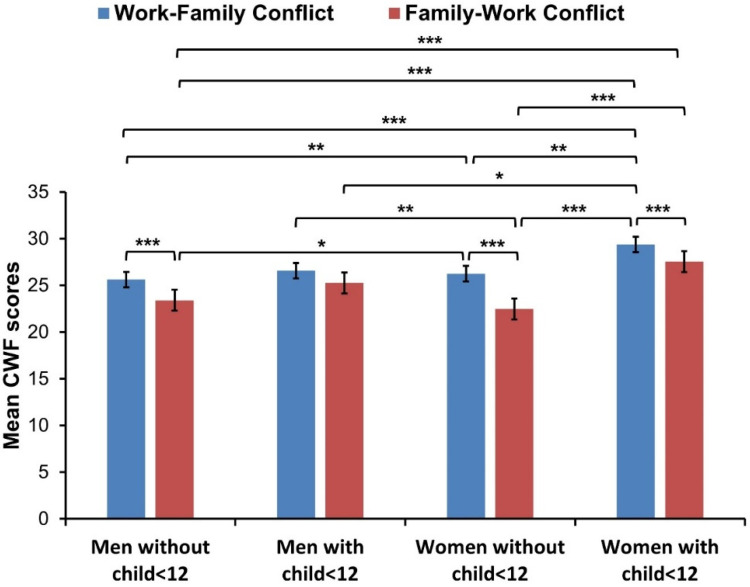
Differences in conflict work-family (CWF) between women and men with and without children under 12. * *p* < 0.05, ** *p* < 0.01, *** *p* < 0.001.

The interaction effect of conflict between work and family, gender, and caring for children under 12, was examined using a three-way ANOVA for repeated measures of conflict between work and family (WFC, FWC) as a dependent variable and gender (men, women) and caring for children under 12 (Yes, No) as categorical factors. The results are shown in Table 1 and Figure 1. Significantly higher scores in WFC than FWC were found in the study, with a medium effect size (*p* < 0.001, η^2^*_p_* = 0.116). Although no gender differences were observed, the interaction between CWF and gender was significant, with a medium effect size (*p* < 0.028, η^2^*_p_* = 0.070). Parents of children under 12 scored significantly lower than those without small children, but the effect size was small (*p* < 0.001, η^2^*_p_* = 0.02). The interaction effect between CWF and caring for children under 12 was significant, but the effect size was small (*p* < 0.002, η^2^*_p_* = 0.013). Interaction between gender and parenting children under 12 was significant but effect size marginal (*p* < 0.048, η^2^*_p_* = 0.005). However, a three-way interaction of CWF with gender and parenthood was insignificant. Details of the inter-group differences using the Bonferroni posthoc test are shown in Figure 1. Overall, women with children under 12 differ significantly in both WFC and FWC from women and men without small children. However, no significant differences in WFC and FWC were reported between women and men with children under 12 (Figure 1). 

### 3.3. Associations between Family-Specific, Work-Specific, and Well-Being Dimensions

**Figure 2:**
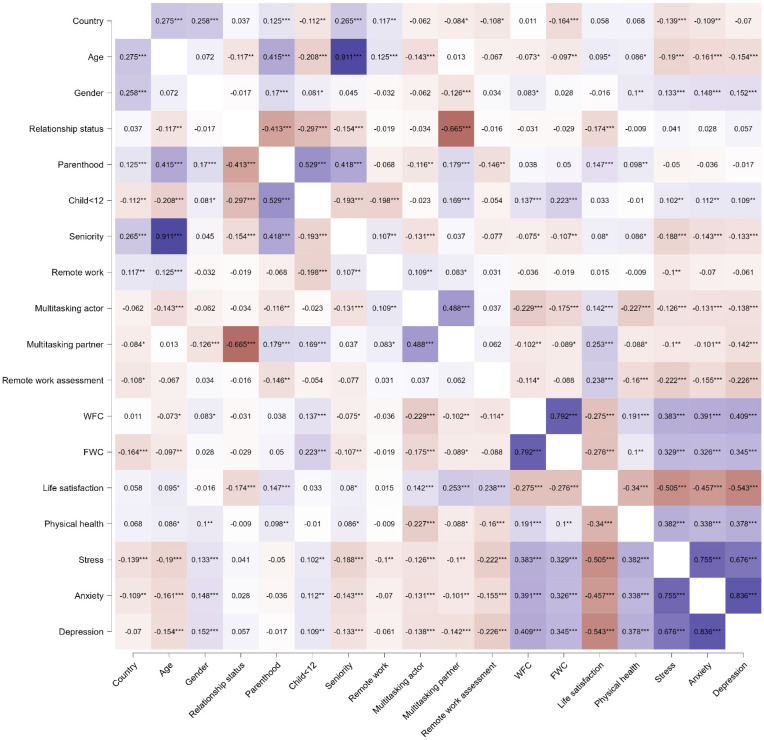
The relationships between the demographic, family-specific and work-specific variables, and well-being dimensions. Positive correlations are marked in violet while negative correlations are marked in red. * *p* < 0.05, ** *p* < 0.01, *** *p* < 0.001.

Associations between all variables were examined using Pearson’s correlations (Figure 2). Life satisfaction is related to older age, coupled relationship status, parenthood status, higher assessment of remote work, and time pressure (both actor and partner), while negatively associated with WFC, FWC, worse physical health, perceived stress, anxiety, and depression. Positive inter-relations were found between variables. such as WFC, FWC, worse physical health, perceived stress, anxiety, and depression. WFC was related to younger age and lower seniority, female gender, caring for children under 12, lower time pressure (both actor and partner), worse assessment of remote work and physical health, and low life satisfaction, while high levels of FWC, perceived stress, anxiety, and depression. Higher FWC was reported in Polish people, those of younger age and lower seniority, parents caring for children under 12, individuals with lower time pressure (actor and partner), and those with worse physical health, low life satisfaction, and high levels of perceived stress, anxiety, and depression. 

The multiple linear regression was performed for WFC and FWC as dependent variables and a set of demographics, family-related, work-related, and well-being variables as predictors. Significant predictors of WFC were: Polish country, low self-perceived time pressure, and high symptoms of stress (see Table 2 for more details). The regression model explained 22% of WFC variance, *R* = 0.47, *R*^2^ = 0.22, *F*(13, 462) = 10.20, *p* < 0.001. The predictor of FWC was caring for children under 12, with a low level of self-perceived time pressure, and high symptoms of stress, as shown in Table 3. The regression model explained 13% of FWC variance, *R* = 0.36, *R*^2^ = 0.13, *F*(13, 462) = 5.25, *p* < 0.001.

### 3.4. The Path Model for Predictors of Life Satisfaction 

**Figure 3:**
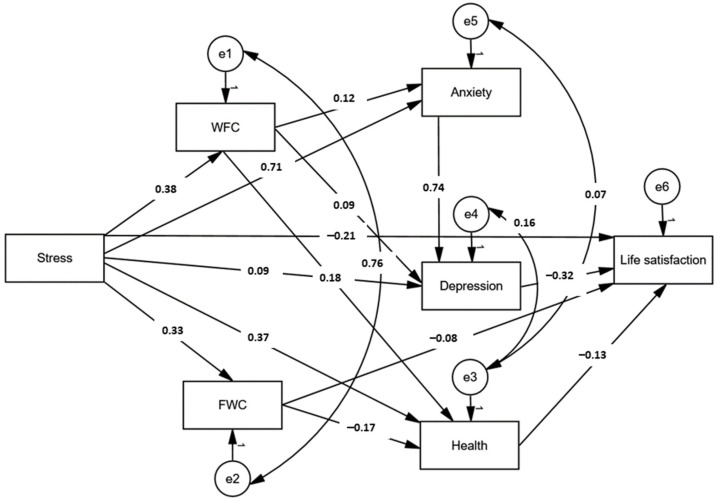
Path model for associations between work-family conflicts and well-being dimensions. All numbers represent statistically significant standardized regression weights (β).

The last step of statistical analysis was developing the path model using SEM. The results are shown in Table 4 and Figure 3. All single-path and mediating effects were significant. Perceived stress increased WFC and FWC, anxiety, and depression and decreased physical health and life satisfaction directly. Both stress and WFC can decrease life satisfaction via a chain of anxiety and depression as mediators. WFC may also contribute to worsening life satisfaction through depression or bad physical health. FWC reduces life satisfaction directly and via better self-rated health. FWC is not related to anxiety or depression. 

The SEM model presented adequate fit statistics, including χ^2^(4) = 11.40, χ^2^/*df* = 2.85, SRMR = 0.010, RMSEA = 0.050, and CFI = 0.997. The configural measurement invariance (MI) was examined across countries for the SEM model. The unconstrained model demonstrated acceptable fit statistics: χ^2^(8) = 25.43, χ^2^/*df* = 3.179, SRMR = 0.013, RMSEA = 0.054, and CFI = 0.994, suggesting the same model structure in Polish and Ukrainian participants. The configural MI was also assumed across genders, χ^2^(8) = 16.79, χ^2^/*df* = 2.10, SRMR = 0.010, RMSEA = 0.039, and CFI = 0.997. The same SEM model structure was valid in single and coupled participants, χ^2^(8) = 16.57, χ^2^/*df* = 2.07, SRMR = 0.018, RMSEA = 0.038, and CFI = 0.997. Equivalence in the pattern of associations between variables was also demonstrated for parents and childless people, χ^2^(8) = 16.56, χ^2^/*df* = 2.07, SRMR = 0.010, RMSEA = 0.038, and CFI = 0.997. Similarly, parents caring for children below 12 years old, and other people, did not differ in the model structure, χ^2^(8) = 23.24, χ^2^/*df* = 2.91, SRMR = 0.012, RMSEA = 0.051, and CFI = 0.995. The configural MI across remote and stationary workers was also found in the study, χ^2^(8) = 14.284, χ^2^/*df* = 1.79, SRMR = 0.013, RMSEA = 0.033, and CFI = 0.998.

## 4. Discussion

### 4.1. Inter-Group Comparisons

This study investigated the associations of WFC and FWC with work-related variables, family-related factors, and well-being dimensions among working people during the second wave of the COVID-19 pandemic in Poland and Ukraine. Numerous inter-group differences were found regarding country, gender, relationship status, parenthood status, and type of work (remote or stationary). In line with previous research [12,13], some country differences were shown in this study. Although WFC did not differ by country, Polish workers scored significantly higher than Ukrainian workers in FWC and perceived their partner’s time pressure alongside higher stress and anxiety. This result may suggest that Poland is more individualistic, while Ukraine is more collectivistic [13]. A recent meta-analysis also showed cross-cultural differences in WFC/FWC [12]. In particular, collectivism moderated the associations between well-being dimensions (job satisfaction, family satisfaction, and life satisfaction) and such predictors, such as hours and demands in work and family areas [12]. Therefore, lower mental health and higher FWC may be related to a higher workload among Polish workers compared to Ukrainian people. Indeed, this study showed that Polish adults spent significantly more time than Ukrainian adults on household activities, such as shopping, cleaning, caring for the elderly or disabled people, repairs and renovations, and stationary work. In contrast, Ukrainian participants were more engaged in social meetings, development, and remote work than people from Poland. The cross-cultural differences in time pressure may explain the higher WFC among Polish adults compared to Ukrainians.

This study showed gender differences, with higher WFC, stress, anxiety, and depression, while also observing a worsening physical health condition among women compared to men. Indeed, the COVID-19 pandemic revealed and deepened gender inequality in various family-related areas of life, as suggested in the review [16]. Consistent with the stereotype and a traditional gender role ideology (GRI) [14,15], women were more engaged than men in childcare and household tasks at the expense of reducing professional work hours and demands during the pandemic [16,19,23,34,35,36,37]. Gender inequality was also shown in an unequal negotiation of space and time in the home during the lockdown when both partners were working from home [56]. Research indicates that men’s workspace and time were prioritized, while women’s were dispersed. Our research is consistent with previous studies since women in this study spent more time on an average day cleaning, cooking, administering childcare, and remote working, compared to men. In contrast, men were more engaged than women in repairs and renovations, learning and training, stationary work, social meetings, entertainment, hobbies, relaxation, and rest.

Previous research found higher WIF and FIW among women than in men in a nationally representative sample of working adults in the United States [34]. Stefanova et al. [43] examined a gender imbalance in the UK, and Ireland residents lived with a partner and worked from home during the pandemic. Women spent significantly more time on caregiving and household activities than their partners. Moreover, caregiving women spent significantly less time at work and had worse self-rated career outcomes than the other groups. However, career outcomes were not predicted by caregiving in men. Furthermore, caregiving duties were significant and positive predictors of work-family conflict in women, but not among men. Additionally, Woodbridge et al. [37] showed that caregiving hours for children directly influenced WFC and FWC among female university staff members in the United States. Lonska et al. [23] showed that among various groups of the Latvian employed population, women under 44 and those with minor children in the household were more likely to face work-life balance difficulties during COVID-19. Miller and Riley [24] indicated that academic mothers experienced bi-directional WFC and FWC during the early stages of the COVID-19 pandemic in the United States. In particular, caring for young children and remote schooling collided with academic work regarding productivity standards, job competency, and commitment. On the other hand, efforts to maintain high academic work requirements interfered with perceived maternal standards, such as providing care and focusing on their children’s achievements. 

As a consequence of a high strain on work-family conflict, a decrease in physical and mental health dimensions among women (including stress, anxiety, and depression symptoms) was reported in this study, as well as in previous research [19,23,24,34,35,36,37,38,39,40,41,42,43]. Both studies by Oakman et al. [41] and Graham et al. [39] showed that Australian working women reported significantly greater WFC and FWC, and concerns about job insecurity, stress, and neck/shoulder pain compared to men. Furthermore, among home-working bank employees from Italy, women showed the greatest concerns for back-to-stationary work, WFC, and workaholism during the pandemic [42]. The number of children was correlated with a higher WFC. Changes in work arrangements during the COVID-19 pandemic also contributed to worsening Japanese working mothers’ mental health and maternal work-life balance, which adversely impacted children’s well-being [36]. 

Relationship status and parenting seem to shed more light on WFC and FWC as well as mental health in the study sample. Indeed, the balance between work and childcare was experienced as a great challenge among parents working remotely at home, especially when schools and other educational institutions were closed. Indeed, this study showed that coupled participants spent significantly more time than singles on childcare, and remote work, while much less on social meetings, hobbies, and development. Compared to childless individuals, parents spent more time on caregiving, and remote work, while they were significantly less engaged in shopping, repairs and renovations, social meetings, entertainment, hobbies, development, relaxation and rest, sleep, stationary work, learning, and training. In particular, parents with children under 12 spent significantly more time on caregiving and were less engaged in social meetings, hobbies, development, learning and training, relaxation, rest, and sleep. Overall, self-perceived time pressure was lower among parents than childless participants, while their partner’s time pressure was perceived to be higher. This result may suggest that caregiving in parents consumed so much time that many other activities, such as maintaining one’s well-being and self-fulfillment, were limited to a minimum or zero. Consequently, parents’ caregiving of children under 12 showed a higher WFC and FWC, along with greater symptoms of mental health disorders, including stress, anxiety, and depression, compared to individuals without small children. 

The ANOVA results did not show gender differences in WFC and FWC in the study sample. In contrast, parents caring for children under 12 experienced significantly higher WFC and FWC than people without small children. However, both WFC and FWC were similarly highly experienced among men and women with children under 12, compared to people of both genders without small children. Furthermore, WFC was significantly higher than FWC in this study. Moreover, sensitivity analysis showed that self-perceived time pressure was more heightened than the partner’s time pressure regarding cleaning, cooking, childcare, and remote work, which may explain the higher WFC than FWC. Consistent with our study, younger children’s ages predicted higher levels of WFC and FWC in Israeli parents during the lockdowns [22]. FWC increased during the COVID-19 pandemic in a sample of employees from Germany, especially among parents and fathers in particular [25]. However, childless participants reported difficulties balancing private life and work demands. The present result is consistent with Graham et al.’s [39] research, which showed that Australian women without children experienced significantly less WFC and FWC than men with children [39]. Hong et al. [40] found increased work overload and parenting stress among female preschool teachers in China during COVID-19. Furthermore, work overload was positively correlated to WFC, and parenting stress to both WFC and FWC. The other study of Chinese working parents showed that maternal FWC was a positive predictor of maternal depressive symptoms, and this association was moderated by undermining cooperation between parents [57]. Paternal FWC was positively correlated to paternal depressive symptoms. However, paternal depressive symptoms were negatively predicted by paternal WFC, with a moderating effect of supporting cooperation. Unfortunately, supportive cooperation between parents was not a significant moderator for mothers. In contrast to previous studies, this result shows that gender inequality does not exist among young families with small children. This may be an inter-generational change in Poland and Ukraine compared to the Australian or Chinese population or a sign of adaptation to family demands among men during the second pandemic wave. Resilience could be a positive change in the following waves of the COVID-19 pandemic.

This study did not find increased WFC and FWC or decreased mental health and well-being dimensions in remote workers, compared to those working stationary, which is in contrast to previous studies [20,21,27,28,29,30,31]. Remote online work demands, stressful acquisition of new technology, poor internet connection during the lockdown, and loss of boundaries between work and family domains, contributed to higher levels of WFC during the pandemic [20,21]. Escudero-Castillo et al. [27] showed worse well-being among teleworkers than those working stationary during the COVID-19 pandemic. Niu et al. [28] showed that people teleworking during the pandemic reported higher WFC than those working stationary or hybrid (partially in office and teleworking) due to increased working and meeting hours. Furthermore, a high WFC, stress, anxiety, depression, and adverse physical symptoms were found in the teleworker’s group [28]. Home-based telework also negatively impacted mental health among a nationally representative sample of Argentinian workers [31]. WFC was associated with occupational stress in Spanish employees working online and stationary, but working hours were related to stress solely among teleworkers [30]. Finally, workload also determined WFC, job-related stress, and job dissatisfaction among constable-ranked police employees from Pakistan [29]. In contrast to previous studies, Polish and Ukrainian remote workers showed significantly lower stress and spent less time than stationary workers on childcare. Still, they had more time for self-development, learning, and training. This research may suggest that people working remotely during the second wave of the pandemic have well adapted to online working, acquired the technical resources and skills, and arranged their job conditions to secure the boundaries between work and family life. However, more research should be performed to verify this assumption.

### 4.2. Associations between Variables

This study suggests that WFC and FWC are related to the worsening of mental health, including high symptoms of stress, anxiety, and depression, and decreased physical health and life satisfaction. The association between WFC/FWC and mental health was also investigated during the COVID-19 pandemic. The result of this study is fully consistent with previous studies [20,22,27,28,31,32,33,58,59,60]. Both WFC and FWC were significantly and positively associated with anxiety and depression, while negatively correlated with life satisfaction among Argentinian teleworkers during the lockdown [31]. Psychological distress was positively associated with WFC and FWC among Israeli workers [22]. The associations between WFC, perceived stress, and posttraumatic stress symptoms were found in Chinese college teachers during the COVID-19 pandemic [58]. WFC was correlated positively with anxiety, while FWC to stress and anxiety among Malaysian university students during the pandemic [60]. Teaching methods and worry about COVID-19 were significant predictors of WFC and FWC among Canadian graduate students [20]. Karakose et al. [59] found that heightened COVID-19 phobia and low life-satisfaction levels predicted high WFC and FWC among Turkish school administrators during the COVID-19 pandemic. Liu et al. [61] showed that risk perception of COVID-19 contributed to nurses’ job withdrawal via WFC during the pandemic. Zou et al. [57] found positive relationships between WFC and depression among Chinese parents. Landolfi et al. [5] indicated that job control, supervisor support, and family support were positive predictors of work-family balance (WFB). In contrast, family workload negatively predicted WFB among Italian schoolteachers during the pandemic. Furthermore, WFB mediated the relationships between these job-related and family-related antecedents and life satisfaction [5].

In addition, WFC was associated with the female gender and a worse assessment of remote work, while higher FWC was found among Polish participants (than Ukrainian participants). WFC and FWC correlated with young age and low seniority, caring for children under 12, and low average time pressure scores (both actor and partner). Parenthood, particularly for minor children, was reported previously as a risk factor for worse WFC/FWC and well-being dimensions [16,18,19,22,23,24]. Young age and seniority are closely related to the early stages of the family structure, which includes parents with children in preschool or early school age. Childcare overload significantly limited the ability to spend time on other activities, which was demonstrated in this study by the low results of time pressure in parents with young children. All associations consistently and consequently indicated that the highest CWF regards mainly young parents with small children. However, the female gender is more related to higher WFC, which is consistent with the results of the gender comparison discussed in the previous section.

The regression analysis showed that among various work-related and family-related variables, and well-being dimensions, the essential predictors of high WFC are: country (Polish participants showed higher WFC than Ukrainian participants), low levels of self-perceived time pressure, and increased symptoms of stress. However, the regression model explained only 22% of the WFC variance, suggesting that the other variables not included in the model may contribute more significantly to the explanation of WFC. The predictors of FWC were: caring for children aged 12 or less, low self-perceived time pressure, and high stress. However, these variables explained even less variance (13%), so more research should be performed to select the other factors of high FWC during the COVID-19 pandemic.

The last part of this study examined the pattern of associations between WFC/FWC and the well-being dimensions. Consistent with the stress-strain model [44] and CMFS [45,46] and previous regression analysis results, stress was a predictor of high WFC and FWC, anxiety, depression, and worse physical health and life satisfaction. Life satisfaction was considered an outcome in the SEM model. As correlation analysis previously showed in this study, life satisfaction was negatively correlated to WFC and FWC, worse physical health, higher perceived stress, anxiety, and depression. However, the path model did not confirm the direct association between WFC and life satisfaction. Otherwise, WFC decreases life satisfaction via worse physical health, higher anxiety, depression, and the anxiety and depression chain. In contrast, FWC can directly contribute to reducing life satisfaction or via physical health as a mediator. The negative association between FWC and physical health suggests that a higher conflict between family and work is related to better health. However, considering previously found associations, since the higher FWC experienced young parents with small children, their overall good health is justified regarding biological and developmental changes during the lifespan. Overall, worse physical health is correlated to life satisfaction. The structure of inter-relations between the CWF, physical and mental health, and life satisfaction, was invariant across Polish and Ukrainian participants, men and women, coupled and single individuals, parents and childless people, those with and without small children, and people working remotely and stationary. Therefore, we can assume that the associations’ structure is universal for working adults. 

### 4.3. Limitation of the Study

The limitations of this study pertain to the snowball sampling method, online recruiting and surveying, and gender imbalance, which may contribute to the bias. Further study should include a representative sample of working adults, more balanced in sociodemographic variables (e.g., age, gender, relationship status, number of children, age of children, socio-economic status) and work-related variables (e.g., employed status, type of job, and workload). The cross-sectional design of this study forces cause-and-effect relationships to be treated with care. Further studies should be performed longitudinally to verify the regression analysis and path model. On the other hand, the advantages of this study are the adequate sample size and the large number of variables included in the models.

## 5. Conclusions

This study evidenced that WFC and FWC bothered working people during the COVID-19 pandemic. For the first time, this study showed a complex model of various associations between work- and family-related variables and well-being dimensions among employees during the pandemic. The most vulnerable group to experience a high WFC are workers from Poland (compared to those from Ukraine), while a high FWC is more likely among parents with children under 12 years of age. Additionally, high perceived stress and low engagement in various activities related to social gatherings, individual development, relaxation, and entertainment can increase WFC and FWC. The consequence of a high CWF is a decrease in mental health and well-being. Furthermore, various paths provide worsening life satisfaction, from heightened stress, via WFC, FWC, physical health, anxiety, and depression.

There are several practical implications for this study. Prevention programs should be implemented to decrease stress, anxiety, and depression and mitigate WFC and FWC. The target groups are women and young parents with small children. It is suggested to support parents’ caregiving of small children by either providing additional options to them or by providing organized programs offered by educational institutions during the COVID-19 pandemic outside the home if the parents are working from home, or by decreasing work demands during the lockdown, letting parents care for their children and reducing the strain between family and work. To improve mental health, in particular, among women, programs focused on implementing the decrease in stress and anxiety, based on coping with stress strategies, meditation, or mindfulness techniques. Additionally, counseling and therapy, and support groups should be provided for the most vulnerable workers. Workplaces should be more involved in helping their employees during a crisis, such as the COVID-19 pandemic, especially during lockdowns.

## Figures and Tables

**Table 1:** A repeated measures two-way ANOVA for conflict work-family.

CWF	Gender	Child < 12	*n*	*M*	*SD*	Effect	*F*(1, 732)	*p*	η^2^*_p_*
WFC	Men	No	170	25.62	8.25	RM CWF	95.68	<0.001	0.116
		Yes	80	26.58	8.52	RM CWF * Gender	4.84	0.028	0.007
	Women	No	290	26.25	8.94	RM CWF * Child < 12	9.31	0.002	0.013
		Yes	196	29.38	9.51	RM CWF * Gender * Child < 12	1.19	0.277	0.002
FWC	Men	No	170	23.39	8.54	Gender	426.39	0.078	0.004
		Yes	80	25.26	7.95	Child < 12	2254.19	<0.001	0.022
	Women	No	290	22.48	8.27	Gender * Child < 12	537.24	0.048	0.005
		Yes	196	27.55	9.39				

Note. CWF = conflict between work and family, WFC = work-family conflict, FWC = family-work conflict, RM = repeated measures factor.

**Table 2:** Multiple linear regression for work-family conflict.

			95% CI					95% CI	
Predictor	*B*	*SE B*	LL	UL	*t*	*p*	β	LL	UL
Intercept	26.22	3.98	18.40	34.03	6.59	<0.001			
Country (1–0)	1.94	0.85	0.28	3.61	2.30	0.022	0.22	0.03	0.41
Gender (1–0)	−1.01	0.87	−2.72	0.71	−1.15	0.249	−0.11	−0.31	0.08
Relationship status (1–0)	−1.85	1.01	–3.83	0.12	−1.84	0.066	−0.21	−0.44	0.01
Parenthood (1–0)	0.21	1.13	–2.02	2.43	0.18	0.857	0.02	–0.23	0.28
Child caregiving < 12 (1–0)	0.55	1.00	−1.41	2.50	0.55	0.582	0.06	−0.16	0.29
Remote working	−0.40	0.92	−2.21	1.40	−0.44	0.660	−0.05	−0.25	0.16
Seniority	−0.05	0.04	−0.14	0.03	−1.22	0.222	−0.07	−0.17	0.04
Remote work assessment	−0.01	0.05	−0.11	0.09	−0.17	0.867	−0.01	−0.09	0.08
Time pressure actor	−0.18	0.04	−0.26	−0.10	−4.34	<0.001	−0.19	−0.27	−0.10
Life satisfaction	0.00	0.08	−0.15	0.15	−0.02	0.980	0.00	−0.10	0.10
Physical health	−0.26	0.28	−0.81	0.30	−0.91	0.364	−0.04	−0.14	0.05
Perceived stress	0.35	0.07	0.20	0.49	4.71	<0.001	0.30	0.17	0.42
Depression	0.20	0.09	0.02	0.38	2.14	0.033	0.13	0.01	0.25

Note. CI = confidence interval, LL = lower level, UL = upper level.

**Table 3:** Multiple linear regression for family-work conflict.

			95% CI					95% CI	
Predictor	*B*	*SE B*	LL	UL	*t*	*p*	β	LL	UL
Intercept	26.37	3.85	18.81	33.94	6.85	<0.001			
Country (1–0)	0.03	0.82	−1.58	1.64	0.04	0.969	0.00	−0.20	0.20
Gender (1–0)	−0.95	0.84	−2.61	0.71	−1.12	0.263	−0.12	−0.33	0.09
Relationship status (1–0)	−0.67	0.97	−2.58	1.24	−0.69	0.492	−0.08	−0.32	0.16
Parenthood (1–0)	−0.83	1.10	−2.98	1.33	−0.75	0.452	−0.10	−0.37	0.17
Child caregiving < 12 (1–0)	2.23	0.96	0.34	4.12	2.31	0.021	0.28	0.04	0.51
Remote working	0.95	0.89	−0.80	2.70	1.07	0.286	0.12	−0.10	0.34
Seniority	0.00	0.04	−0.08	0.08	0.03	0.975	0.00	−0.11	0.11
Remote work assessment	−0.02	0.05	−0.12	0.08	−0.46	0.644	−0.02	−0.11	0.07
Time pressure actor	−0.13	0.04	−0.21	−0.05	−3.21	0.001	−0.15	−0.24	−0.06
Life satisfaction	−0.08	0.07	−0.22	0.07	−1.04	0.298	−0.06	−0.17	0.05
Physical health	−0.54	0.27	−1.07	0.00	−1.96	0.051	−0.10	−0.20	0.00
Perceived stress	0.32	0.07	0.18	0.46	4.49	<0.001	0.30	0.17	0.43
Depression	−0.03	0.09	−0.20	0.15	−0.29	0.774	−0.02	−0.15	0.11

Note. CI = confidence interval, LL = lower level, UL = upper level.

**Table 4:** The path model for life satisfaction (*N* = 736).

			BC 95% CI				
Paths	*B*	*SE*	LL	UL	β	*z*	*p*
PSS ⇒ WFC	0.47	0.04	0.39	0.56	0.38	10.70	<0.001
PSS ⇒ FWC	0.40	0.04	0.31	0.49	0.33	8.98	<0.001
PSS ⇒ GAD	0.55	0.02	0.51	0.58	0.71	32.01	<0.001
WFC ⇒ GAD	0.07	0.02	0.04	0.11	0.12	4.26	<0.001
PSS ⇒ PHQ	0.08	0.03	0.03	0.14	0.09	3.06	0.002
GAD ⇒ PHQ	0.85	0.04	0.77	0.92	0.73	21.31	<0.001
WFC ⇒ PHQ	0.06	0.02	0.03	0.10	0.09	3.83	<0.001
PSS ⇒ GSRH	0.08	0.01	0.06	0.09	0.36	8.39	<0.001
WFC ⇒ GSRH	0.03	0.01	0.01	0.05	0.18	3.32	<0.001
FWC ⇒ GSRH	−0.03	0.01	−0.05	−0.01	−0.17	−3.27	0.001
PSS ⇒ SWLS	−0.18	0.04	−0.25	−0.11	−0.21	−5.03	<0.001
PHQ ⇒ SWLS	−0.31	0.04	−0.39	−0.23	−0.32	−7.96	<0.001
FWC ⇒ SWLS	−0.06	0.03	−0.12	−0.01	−0.08	−2.12	0.034
GSRH ⇒ SWLS	−0.54	0.15	−0.80	−0.21	−0.13	−3.73	<0.001
PSS ⇒ WFC ⇒ GAD ⇒ PHQ ⇒ SWLS	−0.01	0.00	−0.02	−0.01	−0.01	−3.31	<0.001
PSS ⇒ WFC ⇒ PHQ ⇒ SWLS	−0.01	0.00	−0.02	−0.01	−0.01	−3.23	0.001
PSS ⇒ WFC ⇒ GSRH ⇒ SWLS	−0.01	0.00	−0.02	0.00	−0.01	−2.41	0.016
PSS ⇒ FWC ⇒ GSRH ⇒ SWLS	0.01	0.00	0.00	0.01	0.01	2.27	0.023
PSS ⇒ FWC ⇒ SWLS	−0.02	0.01	−0.05	0.00	−0.03	−1.98	0.048
PSS ⇒ GAD ⇒ PHQ ⇒ SWLS	−0.14	0.02	−0.19	−0.10	−0.17	−6.95	<0.001
PSS ⇒ PHQ ⇒ SWLS	−0.03	0.01	−0.05	−0.01	−0.03	−2.91	0.004
PSS ⇒ GSRH ⇒ SWLS	−0.04	0.01	−0.07	−0.02	−0.05	−3.43	<0.001
WFC ⇒ GAD ⇒ PHQ ⇒ SWLS	−0.02	0.01	−0.03	−0.01	−0.03	−3.64	<0.001
WFC ⇒ PHQ ⇒ SWLS	−0.02	0.01	−0.03	−0.01	−0.03	−3.48	<0.001
WFC ⇒ GSRH ⇒ SWLS	−0.02	0.01	−0.03	−0.01	−0.02	−2.50	0.012
GAD ⇒ PHQ ⇒ SWLS	−0.26	0.04	−0.34	−0.19	−0.24	−7.13	<0.001
FWC ⇒ GSRH ⇒ SWLS	0.02	0.01	0.01	0.03	0.02	2.41	0.016

Note. WFC = work-family conflict, FWC = family-work conflict, PSS = perceived stress scale (stress), GSRH = general self-rated health (physical health), GAD = general anxiety disorder (anxiety), PHQ = patient health questionnaire (depression), SWLS = satisfaction with life scale (life satisfaction), BC = bias-corrected percentile bootstrapping, CI = confidence interval, LL = lower level, UL = upper level.

## Data Availability

The data are available from the corresponding author on reasonable request.

## Supplementary Materials

The following supporting information can be downloaded at: https://www.mdpi.com/article/10.3390/ijerph191710954/s1, Table S1: Country differences in work-specific and well-being variables; Table S2: Gender differences in work-specific and well-being variables; Table S3: Relationship status differences in work-specific and well-being variables; Table S4: Parenthood differences in work-specific and well-being variables; Table S5: Caregiving children under 12 differences in work-specific and well-being variables; Table S6: Remote work differences in work-specific and well-being variables; Table S7: Mann–Whitney *U*-test for perceived self-multitasking by country; Table S8: Mann–Whitney *U*-test for perceived self-multitasking by gender; Table S9: Mann–Whitney *U*-test for perceived self-multitasking by relationship status; Table S10: Mann–Whitney *U*-test for perceived self-multitasking by parenthood; Table S11: Mann–Whitney *U*-test for perceived self-multitasking by caregiving children under 12; Table S12: Mann–Whitney *U*-test for perceived self-multitasking by type of work; Table S13: Kruskal–Wallis *T*-test for differences between assessment of actor and partner multitasking.
