# Supplementary material for: Associations of Work-Family Conflict with Family-Specific, Work-Specific, and Well-Being-Related Variables in a Sample of Polish and Ukrainian Adults during the Second Wave of the COVID-19 Pandemic: A Cross-Sectional Study
Source: Int J Environ Res Public Health. 2022 Sep 2;19(17):10954. doi: 10.3390/ijerph191710954 (PMC9517904; doi:10.3390/ijerph191710954)
Supplement: Supplementary file 1 [file ijerph-19-10954-s001.zip › ijerph-1888574-supplementary.pdf]

SUPPLEMENTARY MATERIALS

Table S1. Country differences in work-specific and well-being variables.

Variable	Poland $n = 392$		Ukraine $n = 344$		t(734)	p	d
	M	SD	M	SD			
WFC	26.88	8.95	27.08	9.07	–0.30	0.763	–0.02
FWC	25.70	9.22	22.79	8.15	4.54$_a$	< 0.001	0.33
Remote work	36.59	7.83	34.80	7.22	2.36	0.019	0.24
Time pressure actor	28.26	10.41	27.05	8.88	1.70$_a$	0.091	0.13
Time pressure partner	19.99	13.55	17.76	12.69	2.29	0.022	0.17
Life satisfaction	22.55	6.52	23.28	6.01	–1.58	0.115	–0.12
Physical health	5.33	1.52	5.54	1.48	–1.86	0.064	–0.14
Perceived stress	19.81	6.89	17.79	7.56	3.78$_a$	< 0.001	0.28
Anxiety	8.38	5.69	7.16	5.47	2.97	0.003	0.22
Depression	8.81	6.97	7.88	6.03	1.93$_a$	0.054	0.14

Note. WFC = work-family conflict, FWC = Family-work conflict, a = Welch's *t*-test, *d* = Cohen's *d*.

Table S2. Gender differences in work-specific and well-being variables.

Variable	Men $n = 250$		Women $n = 486$		t(734)	p	d
	M	SD	M	SD			
WFC	25.93	8.33	27.51	9.29	–2.34$_a$	0.020	–0.18
FWC	23.99	8.39	24.52	9.08	–0.77	0.441	–0.06
Remote work	34.87	7.53	35.45	7.40	–0.75	0.456	–0.08
Time pressure actor	28.53	9.88	27.26	9.65	1.68	0.094	0.13
Time pressure partner	21.26	14.08	17.75	12.57	3.44	< 0.001	0.27
Life satisfaction	23.03	6.19	22.82	6.35	0.44	0.658	0.03
Physical health	5.22	1.52	5.53	1.49	–2.72	0.007	–0.21
Perceived stress	17.52	7.15	19.56	7.25	–3.63	< 0.001	–0.28
Anxiety	6.65	5.16	8.41	5.76	–4.20$_a$	< 0.001	–0.32
Depression	6.98	5.98	9.09	6.73	–4.33$_a$	< 0.001	–0.33

Note. WFC = work-family conflict, FWC = Family-work conflict, a = Welch's *t*-test, *d* = Cohen's *d*.

Table S3. Relationship status differences in work-specific and well-being variables.

Variable	Coupled $n = 581$		Single $n = 155$		t(734)	p	d
	M	SD	M	SD			
WFC	27.12	9.17	26.43	8.34	0.85	0.396	0.08
FWC	24.48	8.88	23.84	8.73	0.80	0.425	0.07
Remote work	35.36	7.53	35.07	7.09	0.34	0.735	0.04
Time pressure actor	27.87	9.87	27.05	9.22	0.93	0.355	0.08
Life satisfaction	23.45	6.03	20.77	6.83	4.78	< 0.001	0.43
Physical health	5.43	1.46	5.40	1.67	0.22$_a$	0.828	0.02
Perceived stress	18.71	7.12	19.44	7.83	–1.11	0.269	–0.10
Anxiety	7.73	5.62	8.12	5.62	–0.76	0.445	–0.07
Depression	8.18	6.55	9.10	6.55	–1.55	0.122	–0.14

Note. WFC = work-family conflict, FWC = Family-work conflict, a = Welch's *t*-test, *d* = Cohen's *d*.

SUPPLEMENTARY MATERIALS

Table S4. Parenthood differences in work-specific and well-being variables.

Variable	Parents $n = 346$		Childless $n = 130$		$t(734)$	p	d
	M	SD	M	SD			
WFC	27.21	9.25	26.47	8.44	1.07a	0.287	0.08
FWC	24.64	9.06	23.70	8.37	1.34	0.179	0.11
Remote work	34.64	7.44	37.06	7.15	–3.20	0.001	–0.33
Time pressure actor	26.93	9.75	29.34	9.53	–3.15	0.002	–0.25
Time pressure partner	20.55	11.68	15.49	15.43	4.46a	< 0.001	0.37
Life satisfaction	23.52	6.16	21.54	6.37	4.01	< 0.001	0.32
Physical health	5.53	1.44	5.21	1.61	2.56a	0.011	0.21
Perceived stress	18.62	7.13	19.39	7.57	–1.35	0.178	–0.11
Anxiety	7.67	5.53	8.10	5.81	–0.97	0.335	–0.08
Depression	8.30	6.64	8.54	6.38	–0.47	0.642	–0.04

Note. WFC = work-family conflict, FWC = Family-work conflict, a = Welch's *t*-test, *d* = Cohen's *d*.

Table S5. Caregiving children under 12 differences in work-specific and well-being variables.

Variable	Without Children < 12 $n = 332$		With Children < 12 $n = 144$		$t(734)$	p	d
	M	SD	M	SD			
WFC	26.02	8.69	28.57	9.31	–3.75	< 0.001	–0.29
FWC	22.82	8.37	26.88	9.04	–6.19	< 0.001	–0.47
Remote work	35.56	7.38	34.69	7.54	1.18	0.238	0.12
Time pressure actor	27.87	8.97	27.41	10.92	0.59a	0.559	0.05
Time pressure partner	17.22	13.64	21.82	11.89	–4.80a	< 0.001	–0.36
Life satisfaction	22.73	6.15	23.16	6.53	–0.89	0.373	–0.07
Physical health	5.44	1.45	5.41	1.59	0.27	0.786	0.02
Perceived stress	18.29	7.47	19.83	6.86	–2.79	0.005	–0.21
Anxiety	7.32	5.43	8.62	5.84	–2.99a	0.003	–0.23
Depression	7.82	5.99	9.30	7.33	–2.83a	0.005	–0.22

Note. WFC = work-family conflict, FWC = Family-work conflict, a = Welch's *t*-test, *d* = Cohen's *d*.

Table S6. Remote work differences in work-specific and well-being variables.

Variable	Remote Work $n = 530$		Stationary Work $n = 206$		$t(734)$	p	d
	M	SD	M	SD			
WFC	26.77	8.97	27.49	9.09	–0.97	0.335	–0.08
FWC	24.24	8.85	24.62	8.86	–0.52	0.601	–0.04
Remote work	35.42	7.32	34.83	7.90	0.68	0.494	0.08
Time pressure actor	28.36	9.76	25.99	9.49	2.98	0.003	0.24
Time pressure partner	19.62	13.29	17.19	12.81	2.25	0.025	0.19
Life satisfaction	22.95	6.23	22.74	6.47	0.41	0.686	0.03
Physical health	5.42	1.47	5.45	1.60	–0.24	0.811	–0.02
Perceived stress	18.41	7.29	20.03	7.14	–2.73	0.006	–0.22
Anxiety	7.56	5.66	8.44	5.48	–1.91	0.057	–0.16
Depression	8.13	6.46	9.02	6.78	–1.66	0.098	–0.14

Note. WFC = work-family conflict, FWC = Family-work conflict, *d* = Cohen's *d*.

SUPPLEMENTARY MATERIALS

Table S7. Mann-Whitney *U*-test for perceived self-multitasking by country.

Multitasking	Poland (n = 392)		Ukraine (n = 344)		U	p	Effect Size
	M	SD	M	SD			
Shopping	1.72	1.05	1.52	0.90	75577.00	0.002	0.12
Cleaning	2.13	1.22	1.80	1.00	77116.50	< 0.001	0.14
Cooking	2.34	1.39	2.32	1.16	65886.50	0.580	–0.02
Childcare	1.94	2.07	1.64	1.84	71791.50	0.109	0.07
Elderly/disabled care	0.63	1.13	0.45	1.00	73132.50	0.013	0.09
Repairs, renovations	0.85	1.25	0.40	0.85	82213.00	< 0.001	0.22
Social meetings	1.13	1.58	1.24	1.25	58136.50	< 0.001	–0.14
Entertainment	1.46	1.54	0.53	1.06	93984.50	< 0.001	0.39
Hobbies	1.68	1.49	1.45	1.39	73324.50	0.035	0.09
Development	1.29	1.32	1.76	1.32	52032.00	< 0.001	–0.23
Relax, rest	2.02	1.50	2.13	1.52	64238.50	0.256	–0.05
Sleep	4.06	1.65	4.29	1.43	63023.00	0.050	–0.07
Stationary work	2.80	2.28	2.35	2.01	74743.50	0.008	0.11
Remote work	2.35	2.30	3.59	1.55	48086.00	< 0.001	–0.29
Learning, training	1.86	1.82	1.59	1.51	71253.50	0.171	0.06

Note. Effect size was assessed by rank biserial correlation.

Table S8. Mann-Whitney *U*-test for perceived self-multitasking by gender.

Multitasking	Men (n = 250)		Women (n = 486)		U	p	Effect Size
	M	SD	M	SD			
Shopping	1.60	0.94	1.64	1.01	61165.00	0.869	0.01
Cleaning	1.68	1.02	2.13	1.16	47323.00	< 0.001	–0.22
Cooking	1.84	1.24	2.58	1.23	40096.00	< 0.001	–0.34
Childcare	1.30	1.76	2.06	2.02	48095.00	< 0.001	–0.21
Elderly/disabled care	0.41	0.82	0.61	1.18	57530.00	0.139	–0.05
Repairs, renovations	1.14	1.27	0.38	0.91	87135.50	< 0.001	0.43
Social meetings	1.42	1.54	1.05	1.36	69744.50	< 0.001	0.15
Entertainment	1.54	1.63	0.76	1.21	78333.00	< 0.001	0.29
Hobbies	1.90	1.58	1.40	1.34	71342.50	< 0.001	0.17
Development	1.58	1.33	1.47	1.35	64242.00	0.186	0.06
Relax, rest	2.42	1.58	1.89	1.45	71868.50	< 0.001	0.18
Sleep	4.25	1.46	4.13	1.60	62434.50	0.430	0.03
Stationary work	2.91	2.17	2.43	2.15	69040.50	0.002	0.14
Remote work	2.41	2.17	3.20	1.98	48738.50	< 0.001	–0.20
Learning, training	2.12	1.86	1.53	1.56	71267.00	< 0.001	0.17

Note. Effect size was assessed by rank biserial correlation.

SUPPLEMENTARY MATERIALS

Table S9. Mann-Whitney *U*-test for perceived self-multitasking by relationship status.

Multitasking	Coupled (n = 581)		Single (n = 155)		*U*	*p*	Effect Size
	M	*SD*	*M*	*SD*			
Shopping	1.60	0.97	1.71	1.04	43183.00	0.396	–0.04
Cleaning	1.99	1.16	1.94	1.03	45681.50	0.771	0.02
Cooking	2.37	1.33	2.17	1.10	48264.00	0.154	0.07
Childcare	2.09	1.99	0.70	1.42	63078.50	< 0.001	0.40
Elderly/disabled care	0.54	1.04	0.58	1.21	45781.50	0.687	0.02
Repairs, renovations	0.67	1.15	0.50	0.91	47282.00	0.263	0.05
Social meetings	1.09	1.39	1.51	1.56	37514.00	< 0.001	–0.17
Entertainment	1.02	1.42	1.04	1.39	44414.00	0.776	–0.01
Hobbies	1.53	1.47	1.72	1.34	40124.00	0.032	–0.11
Development	1.46	1.36	1.68	1.24	39390.00	0.013	–0.13
Relax, rest	2.03	1.51	2.24	1.50	41309.00	0.104	–0.08
Sleep	4.16	1.59	4.20	1.43	45843.00	0.657	0.02
Stationary work	2.57	2.16	2.68	2.17	44020.50	0.655	–0.02
Remote work	3.04	2.06	2.52	2.11	51242.00	0.006	0.14
Learning, training	1.70	1.69	1.86	1.67	42048.50	0.193	–0.07

Note. Effect size was assessed by rank biserial correlation.

Table S10. Mann-Whitney *U*-test for perceived self-multitasking by parenthood.

Multitasking	Parents (n = 502)		Childless (n = 234)		*U*	*p*	Effect Size
	M	*SD*	*M*	*SD*			
Shopping	1.58	0.96	1.73	1.02	53471.00	0.034	–0.09
Cleaning	1.94	1.12	2.06	1.15	54854.50	0.130	–0.07
Cooking	2.30	1.25	2.40	1.35	56417.50	0.372	–0.04
Childcare	2.59	1.89	0.12	0.59	102066.50	< 0.001	0.74
Elderly/disabled care	0.58	1.06	0.48	1.11	64210.50	0.010	0.09
Repairs, renovations	0.55	0.99	0.83	1.31	53392.50	0.020	–0.09
Social meetings	0.95	1.24	1.66	1.69	44908.50	< 0.001	–0.24
Entertainment	0.76	1.20	1.60	1.65	40967.00	< 0.001	–0.30
Hobbies	1.35	1.38	2.04	1.49	42178.00	< 0.001	–0.28
Development	1.43	1.30	1.68	1.41	53034.50	0.028	–0.10
Relax, rest	1.83	1.42	2.59	1.57	42618.00	< 0.001	–0.27
Sleep	4.07	1.66	4.40	1.29	53860.50	0.020	–0.08
Stationary work	2.41	2.12	2.98	2.22	49672.50	< 0.001	–0.15
Remote work	3.11	1.99	2.55	2.21	66354.50	0.003	0.13
Learning, training	1.49	1.59	2.24	1.77	44294.00	< 0.001	–0.25

Note. Effect size was assessed by rank biserial correlation.

SUPPLEMENTARY MATERIALS

Table S11. Mann-Whitney *U*-test for perceived self-multitasking by caregiving children under 12.

Multitasking	No Child below 12 (n = 460)		Child below 12 (n = 276)		U	p	Effect Size
	M	SD	M	SD			
Shopping	1.57	0.93	1.72	1.07	59066.00	0.087	–0.07
Cleaning	1.91	1.07	2.09	1.22	59067.00	0.098	–0.07
Cooking	2.31	1.24	2.36	1.35	62000.00	0.583	–0.02
Childcare	0.65	1.23	3.71	1.41	9669.00	< 0.001	–0.85
Elderly/disabled care	0.53	1.13	0.57	0.97	58917.50	0.040	–0.07
Repairs, renovations	0.64	1.12	0.63	1.09	62614.50	0.717	–0.01
Social meetings	1.33	1.48	0.92	1.33	74659.00	< 0.001	0.18
Entertainment	1.12	1.48	0.86	1.28	68377.50	0.055	0.08
Hobbies	1.75	1.43	1.27	1.42	77759.00	< 0.001	0.23
Development	1.71	1.37	1.17	1.22	79149.50	< 0.001	0.25
Relax, rest	2.32	1.50	1.66	1.44	80389.00	< 0.001	0.27
Sleep	4.33	1.35	3.91	1.82	69459.00	0.006	0.09
Stationary work	2.70	2.14	2.40	2.20	68668.50	0.052	0.08
Remote work	3.04	2.04	2.75	2.14	67940.00	0.096	0.07
Learning, training	1.93	1.69	1.41	1.63	75659.00	< 0.001	0.19

Note. Effect size was assessed by rank biserial correlation.

Table S12. Mann-Whitney *U*-test for perceived self-multitasking by type of work.

Multitasking	Remote (n = 530)		Stationary (n = 206)		U	p	Effect Size
	M	SD	M	SD			
Shopping	1.60	0.92	1.68	1.12	53245.50	0.574	–0.03
Cleaning	1.96	1.10	2.02	1.21	53393.50	0.629	–0.02
Cooking	2.38	1.28	2.20	1.29	58420.00	0.126	0.07
Childcare	1.62	1.93	2.26	2.01	44865.00	< 0.001	–0.18
Elderly/disabled care	0.52	1.04	0.62	1.17	53132.50	0.480	–0.03
Repairs, renovations	0.71	1.20	0.45	0.77	58594.00	0.071	0.07
Social meetings	1.21	1.41	1.10	1.50	58934.50	0.076	0.08
Entertainment	1.03	1.44	1.01	1.35	53958.00	0.790	–0.01
Hobbies	1.62	1.45	1.46	1.45	58377.00	0.132	0.07
Development	1.61	1.35	1.23	1.27	64246.00	< 0.001	0.18
Relax, rest	2.13	1.55	1.94	1.39	57751.00	0.210	0.06
Sleep	4.20	1.53	4.11	1.62	56465.00	0.354	0.03
Stationary work	2.26	2.10	3.43	2.10	38293.00	< 0.001	–0.30
Remote work	3.55	1.83	1.35	1.83	86398.00	< 0.001	0.58
Learning, training	1.97	1.73	1.13	1.41	69963.50	< 0.001	0.28

Note. Effect size was assessed by rank biserial correlation.

SUPPLEMENTARY MATERIALS

Table S13. Kruskal-Wallis *T*-test for differences between assessment of actor and partner multitasking.

Multitasking	Actor (n = 581)		Partner (n = 575)		T	Z	p	Effect Size
	M	SD	M	SD				
Shopping	1.60	0.97	1.63	1.13	27289.50	0.57	0.568	0.02
Cleaning	1.99	1.16	1.52	1.27	24587.00	7.07	< 0.001	0.29
Cooking	2.37	1.33	1.57	1.36	25903.00	9.65	< 0.001	0.40
Childcare	2.09	1.99	1.54	1.72	6675.00	8.59	< 0.001	0.36
Elderly/disabled care	0.54	1.04	0.49	1.02	6386.50	1.37	0.170	0.06
Repairs, renovations	0.67	1.15	0.74	1.17	18470.50	1.75	0.081	0.07
Social meetings	1.09	1.39	1.06	1.39	11982.00	0.30	0.766	0.01
Entertainment	1.02	1.42	0.99	1.39	8185.00	0.81	0.415	0.03
Hobbies	1.53	1.47	1.36	1.38	20952.50	2.60	0.009	0.11
Development	1.46	1.36	1.24	1.30	11996.00	4.34	< 0.001	0.18
Relax, rest	2.03	1.51	2.22	1.55	14133.50	3.75	< 0.001	0.16
Sleep	4.16	1.59	4.07	1.64	2839.00	1.67	0.096	0.07
Stationary work	2.56	2.16	2.73	2.26	27486.00	1.18	0.237	0.05
Remote work	3.04	2.06	1.67	2.07	10713.00	11.07	< 0.001	0.46
Learning, training	1.70	1.69	0.91	1.41	9480.00	9.98	< 0.001	0.42

Note. Effect size was assessed by dividing the absolute standardized test statistic *Z* by the square root of the number of compared pairs.
